# Efficacy and safety of 12-hour versus 24-hour magnesium sulfate in management of patients with pre-eclampsia and eclampsia: a systematic review and meta-analysis

**DOI:** 10.1186/s12905-024-03271-0

**Published:** 2024-07-25

**Authors:** Rahma Sameh Shaheen, Rahma Abdelaziz Ismail, Esraa Y. Salama, Sally Median Korini, Ahmed Saad Elsaeidy

**Affiliations:** 1 Faculty of Medicine, Benha University, Benha, Egypt; 2 Faculty of Medicine, Aleppo University, Aleppo, Syria

**Keywords:** Magnesium sulfate, Eclampsia, Pre-eclampsia, Seizure, Pritchard, Zuspan

## Abstract

**Introduction:**

Magnesium sulfate is the most utilized anticonvulsant for treating patients with eclampsia and pre-eclampsia. The purpose of this study is to determine whether the 12-h regimen of magnesium sulfate outweighs the 24-h regimen in both efficacy and safety in the management of patients with mild or severe pre-eclampsia and eclampsia.

**Methods:**

We searched six electronic databases: PubMed, Scopus, Web of Science, Cochrane Library, Ovid, and Google Scholar. This search was conducted to yield any studies that were published until 15 January 2023. We did the statistical analysis plan by Review Manager Software version 5.4.

**Results:**

We included 13 randomized control trials with 2813 patients in this systematic review. Our meta-analysis revealed that there were no statistically significant differences between the 12-h regimen of the magnesium sulfate group and the 24-h regimen of the magnesium sulfate group in our outcome of interest: occurrence of seizure (RD: -0.00, 95% CI [-0.01, 0.00], *P* = 0.56), diminished deep tendon reflexes (RD: -0.00, 95% CI [-0.01, 0.01], *P* = 0.80), respiratory depression (RD: -0.00, 95% CI [-0.02, 0.01], *P* = 0.57), and pulmonary edema (RD: -0.00, 95% CI [-0.01, 0.01], *P* = 0.85).

**Conclusion:**

Our study showed no statistically significant difference in effectiveness and toxicity risk between the 12-h and 24-h regimens.

## Introduction

Pregnancy may be associated with a group of diseases, such as hypertensive disorders of pregnancy, including pre-eclampsia and eclampsia. Both serious medical conditions affect 10% of pregnancies [1]. Pre-eclampsia and eclampsia put maternal health at risk by increasing maternal morbidity and mortality [2, 3].

Pre-eclampsia is considered a multisystem disease that targets many organs [2]. It is characterized by a hypertensive condition that newly affects women with previously normal blood pressure after twenty weeks of pregnancy [4]. It is caused by cell dysfunction and altered trophoblastic invasion [5].

Pre-eclampsia is a medical condition diagnosed when a pregnant woman’s systolic blood pressure reaches 140 mmHg or higher, or her diastolic blood pressure reaches 90 mmHg or higher, along with proteinuria, which is characterized by a urine protein: creatinine ratio of 30 mg/mmol or more, or albumin: creatinine ratio of 8 mg/mmol or more, or at least 1 g/liter [2 +] on dipstick testing. Renal insufficiency may manifest by a creatinine level of 90 micromole/liter or higher, or a serum creatinine level of 1.02 mg/100 ml or higher. Additionally, liver affection is indicated by elevated transaminases with alanine aminotransferase or aspartate aminotransferase levels above 40 IU/L. Liver involvement may present with right upper quadrant or epigastric abdominal pain. Thrombocytopenia (platelet count < 150,000/µL), disseminated intravascular coagulation or hemolysis may manifest as hematological complications. Furthermore, uteroplacental dysfunction may complicate the pre-eclampsia and manifest by fetal growth restriction, abnormal umbilical artery doppler waveform analysis, or stillbirth [6].

On the other hand, severe pre-eclampsia is a medical condition characterized by persistent and severe hypertension in a patient who does not respond to treatment, as well as ongoing or recurring symptoms such as severe headaches, visual disturbances, nausea or vomiting, epigastric pain, oliguria, and worsening laboratory blood tests, including high levels of creatinine, liver transaminases, decreased platelet count, decreased fetal growth, or abnormal doppler findings [6]. It is crucial to consider it a serious complication, as it correlates with a high risk of maternal death before, during, or after delivery [7].

Although eclampsia in Greece is defined as “a flash of lightning,” which may not match its seriousness, it is defined medically as convulsions related to pre-eclampsia without any obvious neurological as a concurrent cause [8]. Regarding pre-eclampsia and eclampsia, their ideal treatment is primarily to maintain the patient stable, control hypertension, prevent or treat seizures, and arrange for delivery, which is considered the definite cure [9, 10].

Among numerous anticonvulsants, magnesium sulphate (MgSO_4_) has been used in clinical practice since 1925. For decades, it has still been the most utilized anticonvulsant for controlling patients with eclampsia and pre-eclampsia [1, 2].

Despite this wide use of MgSO_4_, its exact mechanism is still unclear. However, the most accepted theories suggest that it interferes with calcium homeostasis and controls fits by both neurological and cardiovascular effects [11]. According to the use of MgSO_4_ in both preventing and treating fits, it is recommended to be administered for 24 h after the latest event, whichever delivery or the last fit [3]. Both Pritchard and Zuspan are considered the standard methods for administering MgSO_4_. The Zuspan regimen consists of a loading dosage of 4 g of MgSO_4_ administered IV slowly over five to ten minutes, followed by a maintenance dose of 2 g per hour IV infusion administered for 24 h. The Pritchard regimen consists of a loading dosage of 4 g of MgSO_4_ given IV slowly over five to ten minutes added to concurrent 10 g IM and a maintenance dose of 5 g of MgSO_4_ IM each four hours for 24 h [4, 11, 12].

Although preventing or treating fits, MgSO_4_ administration can cause serious intoxication symptoms like respiratory depression or arrest, absent or minimal deep tendon reflex, and oliguria [5]. Although these serious complications can be considered rare compared to other common, less significant side effects such as flushing, muscle weakness, headache, nausea, vomiting, thirst, confusion, and dizziness, considering these serious intoxication symptoms is still crucial [2]. Based on MgSO_4_ toxicity, the administration of MgSO_4_ demands regular checking of patients by experienced doctors, which is cost-consuming, so reducing the duration of MgSO_4_ administration is expected to be beneficial not only for patients’ health but also for healthcare systems [2, 3].

Although strong evidence indicates magnesium sulfate’s effectiveness in both the prophylaxis and treatment of seizures in pre-eclampsia and eclampsia patients, the ideal duration of MgSo_4_ administration is not yet known. Magnesium sulfate is typically administered for 24 h following delivery in cases of pre-eclampsia. However, more recent research has suggested the potential benefits of shorter duration postpartum MgSo_4_ therapy [10]. Many studies have shown that the incidence of seizures is higher in patients who are receiving 24-h MgSo_4_ [1, 12, 13] while other studies have reported that the incidence of seizures is higher in patients receiving 12-h MgSo_4_ [2, 8, 14]. Therefore, we conducted this systematic review and meta-analysis to assess if 12- hour MgSo_4_ could be a promising alternative to 24-h MgSo_4_ in patients with eclampsia and pre-eclampsia.

## Methods

This systematic review and meta-analysis protocol got the PROSPERO registration ID: CRD42023399554 on 25 February 2023. We conducted this study according to the PRISMA guidelines 2020 [15].

### Search strategy

We searched six electronic databases: PubMed, Scopus, Web of Science, Cochrane Library, Ovid, and Google Scholar. The search was conducted to yield any studies that were published until 15 January 2023, using the search strategy (("12 h") AND ("24 h") AND ("Magnesium sulfate" OR "Heptahydrate Magnesium Sulfate" OR "Epsom Salt" OR "Sulfamag" OR "English salt" OR "Bitter salts" OR "Bath salt" OR "Concept Ob" OR "Suprep Bowel Prep Kit" OR "Tis- U-sol") AND ("Eclampsia" OR ("pre-eclampsia")) and without performing any filter. We manually searched the reference lists of included studies to include other relevant studies that our strategy could not reach.

On 19 September 2023, we updated our search manually and included a new randomized controlled clinical trial.

### Eligibility criteria

The population of interest was pre-eclampsia or eclampsia patients who received no intervention other than a loading dose of MgSO_4_, even if the cases was diagnosed in their antepartum, intrapartum, or postpartum period. We included eclampsia females who had complications such as HELLP syndrome (a condition in pregnant and postpartum individuals characterized by hemolysis with a microangiopathic blood smear, elevated liver enzymes, and a low platelet count), acute renal failure, gestational diabetes, and MgSO_4_ hypersensitivity. We included only the randomized controlled clinical trials which compare between 12 h versus 24 h using MgSo_4_ in managing patients with eclampsia and pre-eclampsia using Pritchard or Zuspan regimen and written in English. Outcomes of interest were the occurrence of seizures, diminished deep tendon reflexes, respiratory depression, and pulmonary edema. Non-original research (i.e., reviews, comments, guidelines, editorials, correspondence, letters to editors, etc.) and any studies that have not compared the effectiveness of 12-h versus 24-h using MgSO_4_ in treatment of patients with eclampsia or pre-eclampsia were excluded. Additionally, studies in a language other than English were excluded. Patients who experienced recurrent attacks of seizures when already on a MgSO_4_ regimen, or who had pre-existing DM, renal disease, anuria/oliguria (urine output < 25 ml/h), and patients who had any contraindication to MgSO_4_ use except drug hypersensitivity (e.g., drug myasthenia gravis) or who experienced coma were excluded.

### Study selection

After searching the databases, all retrieved records were imported into EndNote X9 software, and the duplicate studies were identified and deleted. Two authors conducted the two screening phases separately via Microsoft Excel. Title and abstract screening, then, the full text of eligible articles screening. Any conflict was resolved by a third researcher opinion and discussion for final accessibility to meta-analysis.

### Data extraction

Data extraction was conducted using Microsoft Excel manually by two independent authors and then reviewed by a third author. If there is any disagreement, a meeting between reviewers could be held to resolve it.

Data extracted from the included study were in three groups: 1—General study characteristics (study ID, country at which the study was performed, and sample size). 2—Baseline patient characteristics (age, gestational age, SBP, DBP, and mode of delivery). 3—Outcome measures (seizure occurrence, respiratory depression, diminished deep tendon reflex, and pulmonary edema).

### Quality assessment of included studies

Quality assessment was performed using the Cochrane risk of bias assessment tool by two independent reviewers [16]. Seven domains were studied: sequence generation, allocation sequence concealment, blinding of participants and personnel, blinding of outcome assessment, incomplete outcome data, selective outcome reporting, and other risks of bias, in which judgments (high risk, low risk, or unclear risk) for each item in each trial are presented alongside their descriptive justification. The risk of bias was determined across all outcomes within each included study. A meeting between reviewers could be held in case of any disagreement. Potential publication bias was visually assessed using funnel plots.

### Data synthesis

We conducted the statistical analysis using the Review Manager (RevMan) software program V.5.4. Results with a *P*-value < 0.05 were regarded as significant in the Z-test, and the meta-analysis result for dichotomous outcomes was shown as a risk difference with a 95% confidence interval and conducted by using the fixed-effect model. To assess heterogeneity, we employed the Chi-square and I-square tests, as the Chi-square test assesses the presence of heterogeneity, and the I-square test assesses its degree. The result of the I-square test was interpreted as follows: not significant for 0–40 percent, moderate heterogeneity for 30–60 percent, substantial heterogeneity for 50–90 percent, and considerable heterogeneity for 75–100 percent, following the Cochrane handbook (chapter nine). According to the Chi-square test, we considered an alpha level below 0.1 as a significant heterogeneity.

## Results

### Study selection

**Fig. 1:**
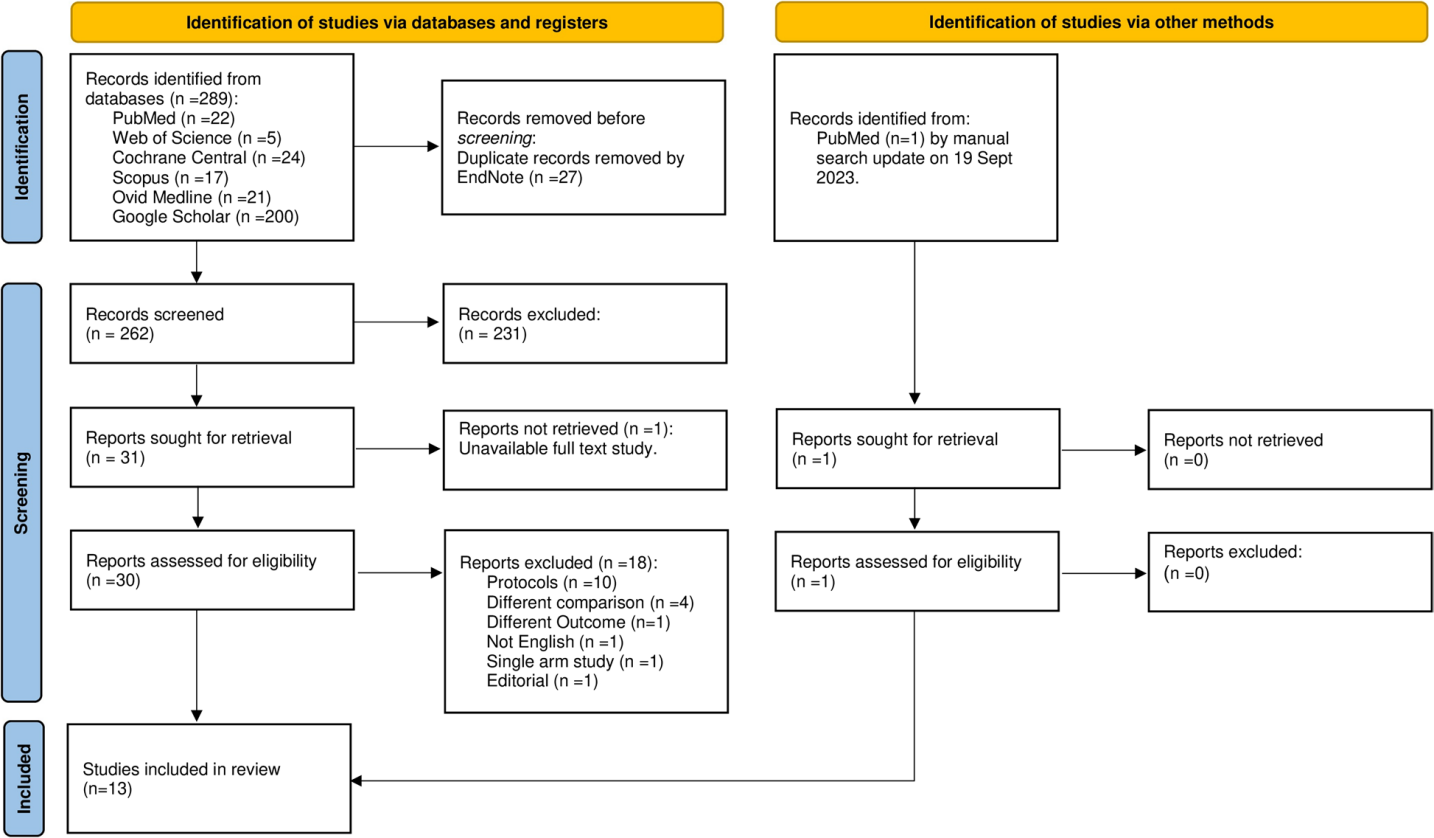
PRISMA flow diagram

By searching the databases, we reached 289 records. After the removal of 27 duplicates by endnote software, the titles and abstracts of 262 studies were screened. So, the full text of 30 studies was screened according to the eligibility criteria. We excluded 18 studies as they did not meet the eligibility criteria. Finally, 13 studies were included in our systematic review as shown in Fig. 1.

### Study characteristics

The summary of the included studies is presented in Table 1. Two studies have been conducted in Egypt [2, 14], three in Nigeria [4, 12, 13], two in India [3, 9], two in Pakistan [11, 17], one in Iran [8], one in Brazil [7], one in Ghana [1], and one in the USA [10]. The current study pools data of 1435 women treated using 12-h MgSO_4_ and 1374 women treated using 24-h MgSO_4_. All included studies were published between 2006 and 2023. In the included studies, one study included patients diagnosed with mild pre-eclampsia [10], seven studies included patients diagnosed with severe pre-eclampsia [2, 4, 7–9, 12, 14], two studies reported the treatment of both patients with eclampsia and pre-eclampsia [1, 13], Three studies included patients with eclampsia [3, 11, 17]. According to our included studies, four studies [1, 8, 9, 12] used the Pritchard regimen, while six studies [3, 4, 10, 11, 13, 17] used the Zuspan regimen. The baseline characteristics of the pre-eclampsia and eclampsia patients are shown in Table 2.

**Table 1:** Summary of the included studies

Study ID	Country	12 h MgSo_4_			24 h MgSo_4_		
**Pre-Eclampsia**		**Sample size**	**Loading dose**	**Maintenance dose**	**Sample size**	**Loading dose**	**Maintenance dose**
Grillo et al. 2023 [13]	Nigeria	74	4 g on 20% in normal saline IV slowly over 20 min	1 g/h IV for 12 h	74	4 g on 20% in normal saline IV slowly over 20 min	1 g/h IV for 24 h
Hekal et al. 2020 [2]	Egypt	80	6 g on 250 ml ringer solutions over 20 min by IV drip	4 g on 250 ml ringer solution over 4 h/4 h by IV drip for 12 h	80	6 g on 250 ml ringer solutions over 20 min by IV drip	4 g on 250 ml ringer solution over 4 h/4 h by IV drip for 24 h
Orisabinone et al. 2020 [12]	Nigeria	58	4 g slow IV bolus + 10 g IM (5g in each buttock)	5 g IM/ 4 h in alternate buttocks for 12 h	58	4 g slow IV bolus, plus 10 g IM (5g in each buttock)	5 g IM/4 h in alternate buttocks for 24 h
Dixit et al. 2020 [9]	India	33	4 g IV + 10 g IM	5g /4 h of a 50% in normal saline IM alternate buttocks for 12 h	34	4g IV + 10 g IM	5g /4 h of a 50% in normal saline IM alternate buttocks for 24 h
Unwaha et al. 2020 [4]	Nigeria	40	4 g on 20% in normal saline IV slowly over 10 min	5 g in 500 mL of normal saline IV 1 g/h for 12 h	40	4 g on 20% in normal saline IV slowly over 10 min	5 g in 500 mL of normal saline IV 1 g/h, for 24 h
Kashanian et al. 2015 [8]	Iran	79	4 g slowly (1 g/m) + 10 g IM	5 g IM/4 h for 12 h	91	4 g slowly (1 g/m) + 10 g IM	5 g IM/4 h for 24 h
El-Khaya et al. 2014 [14]	Egypt	80	6 g on 250 ml ringer solutions by IV drip over 20 min	4 g on 250 ml ringer solution IV drip 1 g/h for 12 h	80	6 g on 250 ml ringer solutions by IV drip over 20 min	4 g on 250 ml ringer solution IV drip 1 g/h for 24 h
Maia et al. 2014 [7]	Brazil	56	6 g	1 g/h IV for 12 h	56	6 g	1 g/h IV for 24 h
Ehrenberg et al. 2006 [10]	USA	101	4 g IV before delivery or within 2 h postpartum	2 g/h IV for 12 h	95	4 g IV before delivery or within 2 h postpartum	2 g/h IV for 24 h
**Eclampsia**							
Beyuo et al. 2022 [1]	Ghana	592	4 g IV and 10g IM (5g.in each buttock)	5 g IM/4 h for 12 h	584	4 g IV and 10g IM (5g.in each buttock)	5 g IM/4 h for 24 h
Khan et al. 2021 [17]	Pakistan	50	4 g IV in 10 min	1 g/h IV for 12 h	50	4 g IV in 10 min	1 g/h IV for 24 h
Anjum et al. 2015 [3]	India	132	4 g IV	1 g/h for 12 h after the last fit or delivery	76	4 g IV	1 g/h for 24 h after the last fit or delivery
Rao et al. 2015 [11]	Pakistan	60	4 g on 20% in normal saline IV slowly over 15 min	1 g (20% in normal saline)/h IV for 12 h	60	4 g (20% in normal saline) IV slowly in 15 min	1 g (20% in normal saline)/h IV for 24 h

*IV* Intravenous infusion, *IM* Intramuscular injection, *min* minute, *h* hour, *g* gram

**Table 2:** Baseline characteristics of the included studies

Study ID	MgSo_4_	Age, y M (SD)		Gestational age, W M (SD)	SBP, mmHg M (SD)		DBP, mmHg M (SD)	Mode Of Delivery	
**Pre-Eclampsia**								**No. Vaginal Delivery**	**No. Cesarean Section**
Grillo et al. 2023^a^ [13]	12 h	29 (8)		38 (3)	170 (30)		110 (20)	15	59
	24 h	30 (10)		37 (5)	170 (23)		110 (30)	53	21
Hekal et al. 2020 [2]	12 h	26.6 (5)		38.2 (2)	162.5 (14.9)		105.1 (10.3)	37	43
	24 h	26.6 (5.2)		38.2 (1.8)	161.1 (16.9)		100.3 (13.9)	35	45
Orisabinone et al. 2020 [12]	12 h	26.7(5.9)		NA	178.1 (19.3)		105.1 (23)	27	31
	24 h	27.7(6.7)		NA	180 (23.3)		98.4 (32.3)	26	32
Dixit et al. 2020 [9]	12 h	27.9 (4.8)		36.7 (2.6)	166.2 (13.3)		104.7 (9.5)	16	16
	24 h	27.3 (5.2)		36.6 (2.7)	165.2 (12.4)		104.1 (10.5)	17	18
Unwaha et al. 2020 [4]	12 h	34.1 (4.5)		34.4 (4)	174.5 (27.7)		110.8 (21.8)	15	25
	24 h	32.3 (6.1)		34.1 (4.5)	175.3 (27.7)		110.9 (15.9)	10	30
Kashanian et al. 2015 [8]	12 h	28.9 (6.1)		36.1 (1.2)	152.2 (12.3)		95.2 (9.4)	NA	NA
	24 h	29.9 (6.1)		36.2 (1.3)	158.3 (15.4)		95.1 (9.5)	NA	NA
El-Khaya et al. 2014 [14]	12 h	26.6 (5)		35.9 (2.9)	162.5 (14.9)		105.1 (10.3)	37	43
	24 h	26.6 (5.2)		35.6 (2.7)	161.1 (16.9)		100.3 (13.9)	35	45
Maia et al. 2014 [7]	12 h	24.7 (6.3)		36.8 (3)	142.4 (16.4)		92.4 (11.9)	20	36
	24 h	26.3 (7.6)		37.2 (4.9)	142.1 (15.1)		95.4 (10.8)	23	33
Ehrenberg et al. 2006 [10]	12 h	24.4 (6.5)		38.7 (1.7)	143 (13)		82 (10)	79	22
	24 h	25.2 (6.5)		38.7 (1.7)	140 (13)		82 (11)	70	25
**Eclampsia**									
Khan et al. 2021 [17]	12 h	≤ 30 y^b^	23 (46%)	36.7 (1.3)	138.2 (11.5)		85.6 (12.4)	15	35
		> 30 y^b^	27 (54%)						
	24 h	≤ 30 y^b^	22 (44%)	36.6 (1.7)	141.5 (11.8)		90 (8.1)	19	31
		> 30 y^b^	28 (56%)						
Anjum et al. 2015 [3]	12 h	23.8 (3.4)		35.6 (1.2)	166.7 (18.8)		102.2 (8.7)	NA	NA
	24 h	24.5 (3.6)		36.5 (1.6)	161.6 (21.5)		100.3 (13.9)	NA	NA
Rao et al. 2015^b^ [11]	12 h	20–24 y	33 (55%)	NA	≤ 90	6 (10%)	NA	NA	NA
					91–100	7 (11.6%)			
		25–29 y	14 (23.3%)		101–110	13 (21.6%)			
		30–40 y	13 (21.6%)		≥ 110	34 (56.6%)			
	24 h	20–24 y	35 (58.3%)	NA	≤ 90	4 (6.6%)	NA	NA	NA
					91–100	9 (15%)			
		25–29 y	10 (16.6%)		101–110	15 (25%)			
		30–40 y	15(25%)		≥ 110	32 (53.3%)			
Beyuo et al. 2022 [1]	12 h	31 (6)		36.5 (4.2)	168.8 (16.3)		107.3 (11.1)	214	369
	24 h	31.3 (5.9)		35.2 (4.4)	165.7 (21.6)		109 (14.2)	172	398

*M (SD)* mean (standard division), *y* year, *w* week, *SBP* Systolic blood pressure, *DBP* Diastolic blood pressure

^a^Data of Grillo et al. [13] reported as median (interquartile range)

^b^Data reported as frequency (percentage)

### Risk of bias

**Fig. 2:**
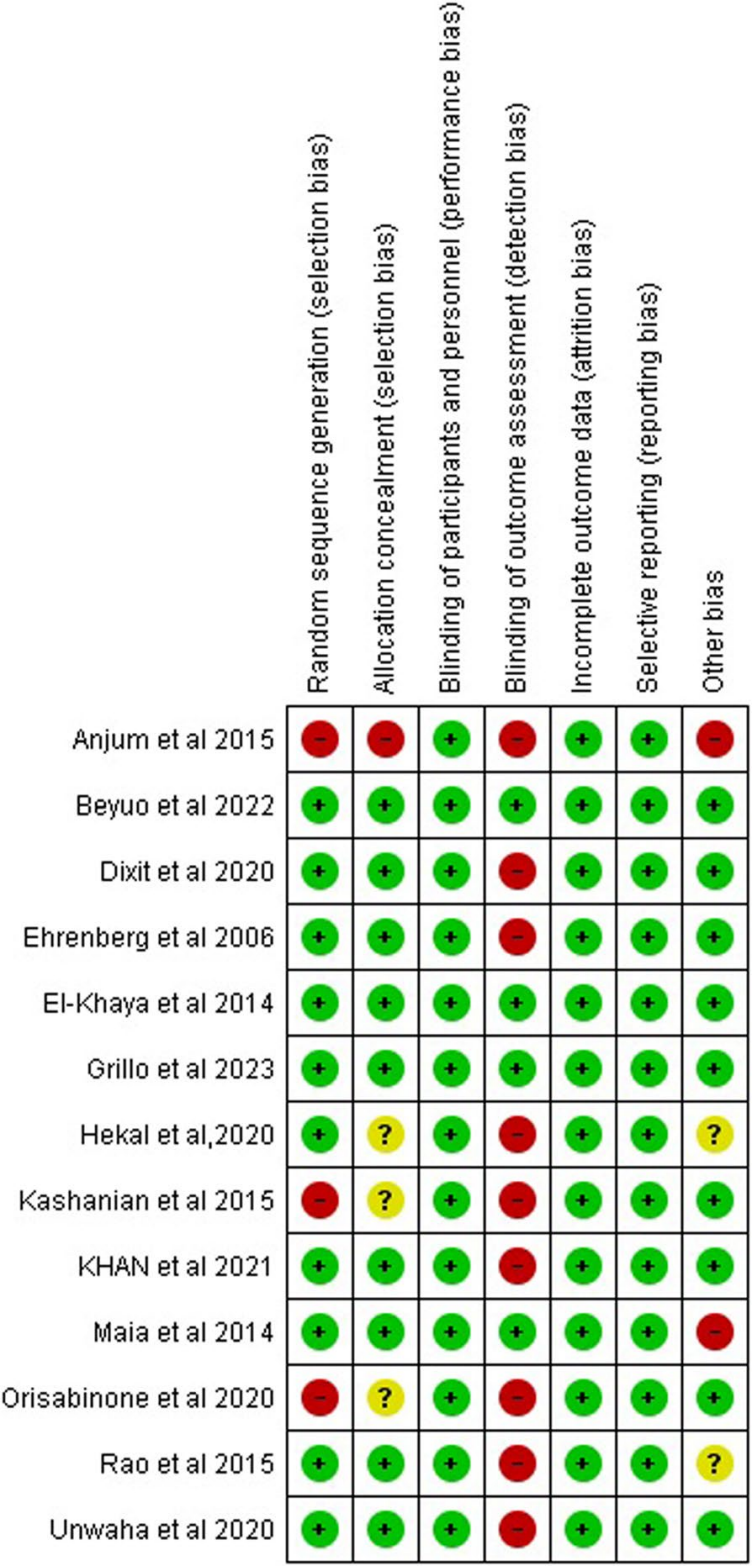
Summary of risk of bias in each study

**Fig. 3:**
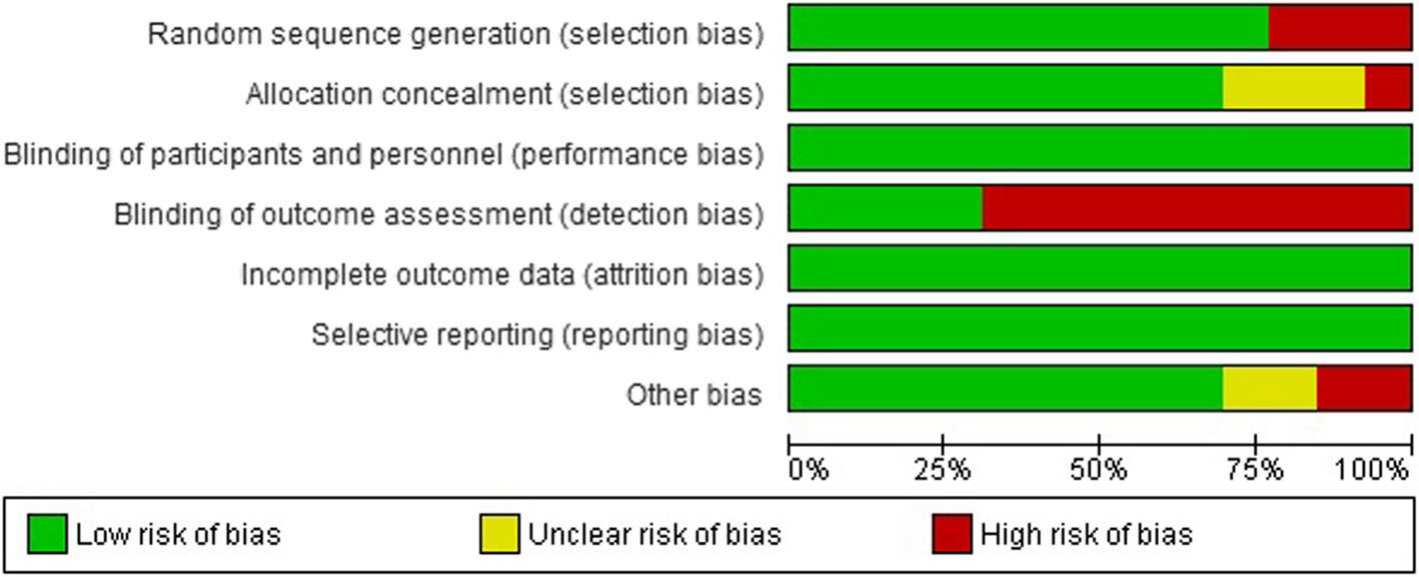
Graph of risk of bias

The summary of the quality assessment of the included studies is presented in Figs. 2 and 3. Three studies [7, 13, 14] have a low bias in all domains. Three studies have a high risk of bias in the random sequence generation as they did not mention any information about the randomization process [3, 8, 12]. In allocation concealment, Anjum et al. [3] have a high risk of bias as the data analysts and investigators were not blinded to group assignment, three studies [2, 8, 12] did not mention enough information about the process of concealment, the other studies have a low risk of bias. In the binding outcome assessment, four studies have a low risk of bias [1, 7, 13, 14]. All studies have a low risk of bias in blinding participants and personnel, incomplete outcome data, and selective reporting. Finally, The last domain was low risk of bias in all studies except two [2, 11] as there was insufficient information to determine whether an important risk of bias exists.

### Occurrence of seizure

We found that seizures had occurred in six pre-eclampsia patients on 12-h MgSo_4_ versus eight patients on 24-h MgSo_4._ On the other hand, in eclampsia patients, seizures occurred in two patients on 12-h MgSo_4_ and two patients on 24-h MgSo_4_. However, we found no seizure risk difference between both MgSo_4_ regime groups.

**Fig. 4:**
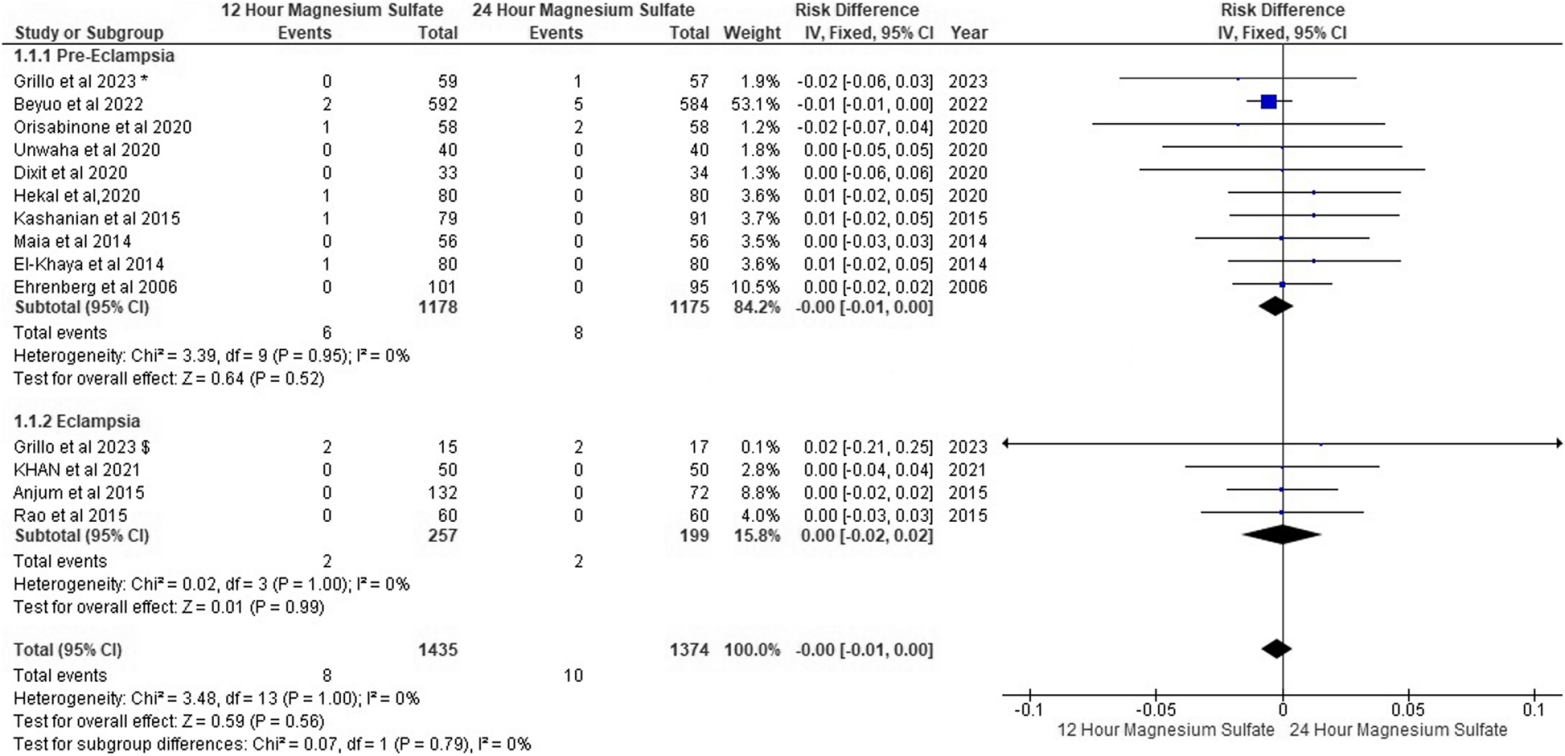
Occurrence of seizures forest plot

**Fig. 5:**
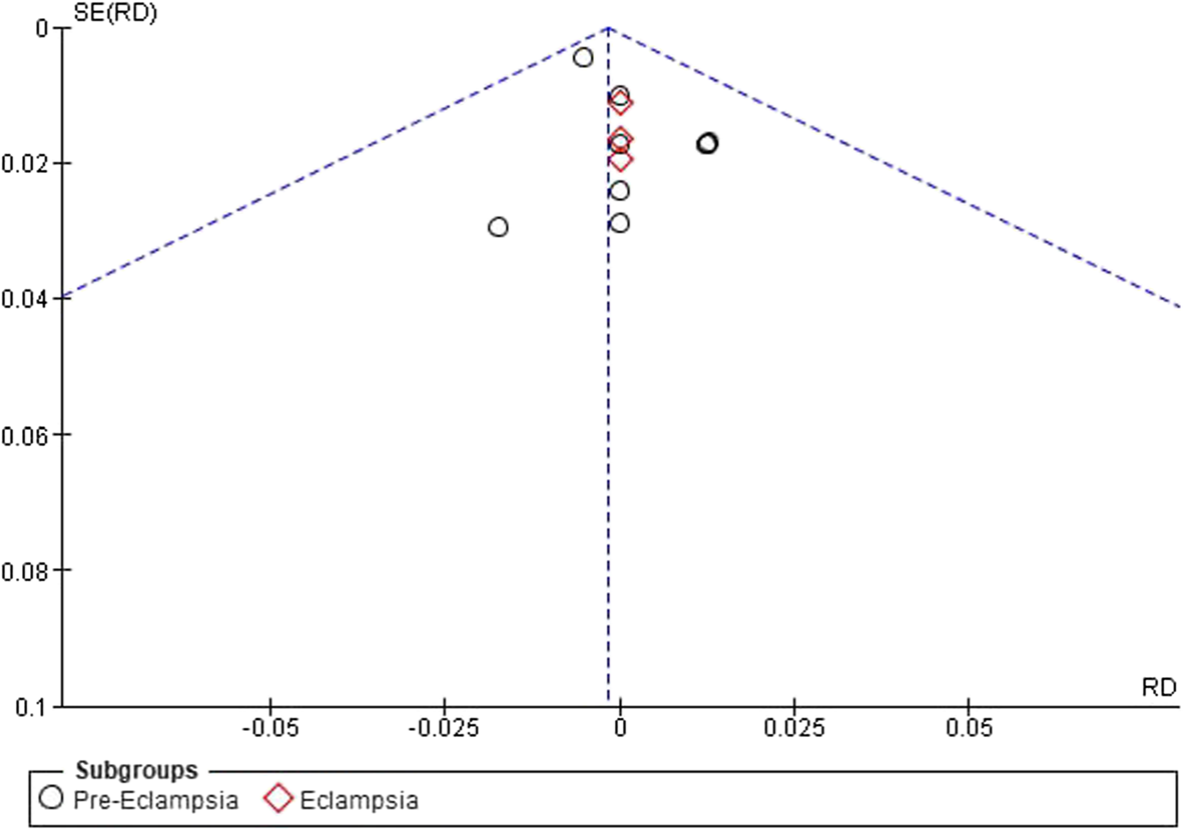
Occurrence of seizures funnel plot

For pre-eclampsia, the pooled risk difference was (RD: -0.00, 95% CI [-0.01, 0.00], *P* = 0.52), and the pooled risk difference for eclampsia was (RD: -0.00, 95% CI [-0.02, 0.02], *P* = 0.99). The pooled results were homogeneous in both subgroups (*P* = 0.95, I^2^: 0%) and (*P* = 1.00, I^2^: 0%) Fig. 4. Additionally, funnel plots and Egger’s test found no evidence of publication bias, as shown in Fig. 5.

### Diminished deep tendon reflexes

**Fig. 6:**
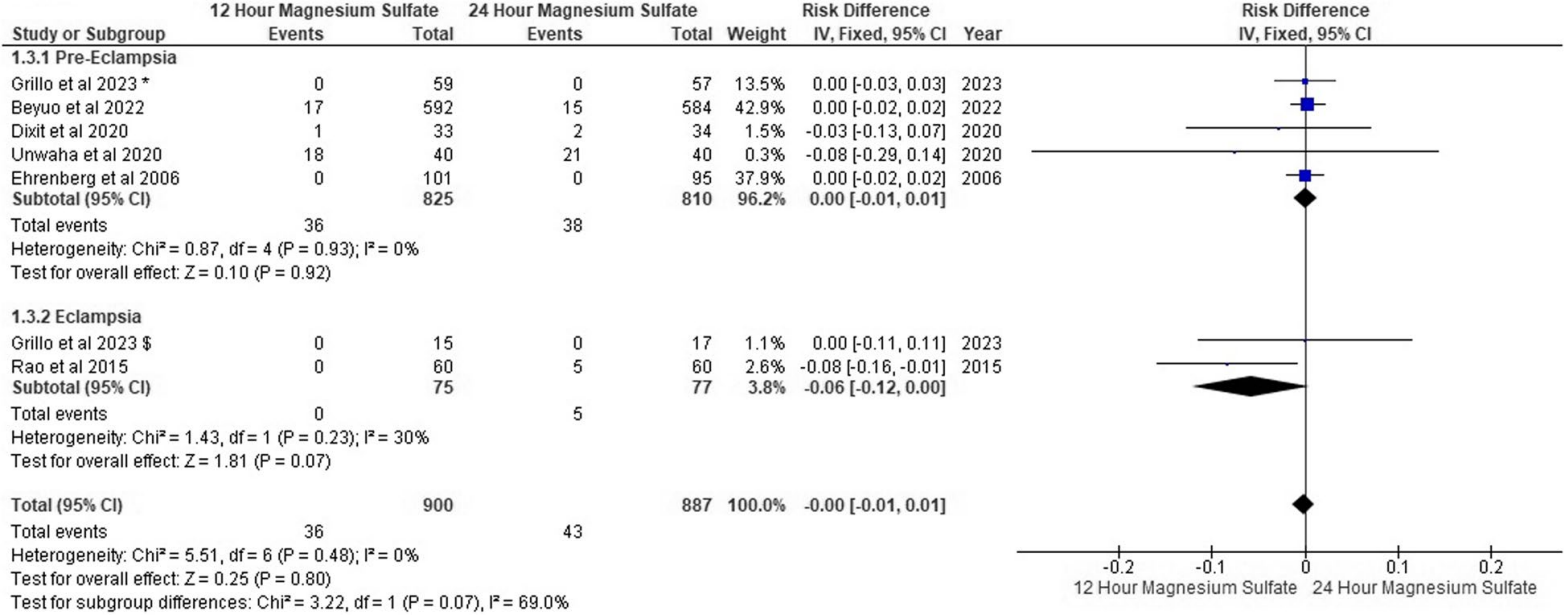
Diminished deep tendon reflexes forest plot

Five trials of pre-eclampsia reported that 36 patients in the 12-h MgSo_4_ group were complicated with diminished deep tendon reflexes versus 38 patients in the 24-h MgSo_4_ group. Also, eclampsia trials showed that five patients were complicated with diminished deep tendon reflexes in the 24-h MgSo_4_ group, although no patients were affected in the 12-h MgSo_4_ group. We found no risk difference between both MgSo_4_ regimen groups with overall pooled risk difference (RD: -0.00, 95% CI [-0.01, 0.01], *P* = 0.48) with no heterogeneity (*P* = 0.48, I^2^: 0%) as shown in Fig. 6.

### Respiratory depression

Four studies of pre-eclampsia reported no respiratory depression in both groups. However, Beyuo et al. [1] reported that 27 patients were complicated with respiratory depression in 12-h MgSo_4_ versus 35 patients in 24-h MgSo_4_. According to Grillo et al. [13], no eclamptic patients were complicated with respiratory depression.

**Fig. 7:**
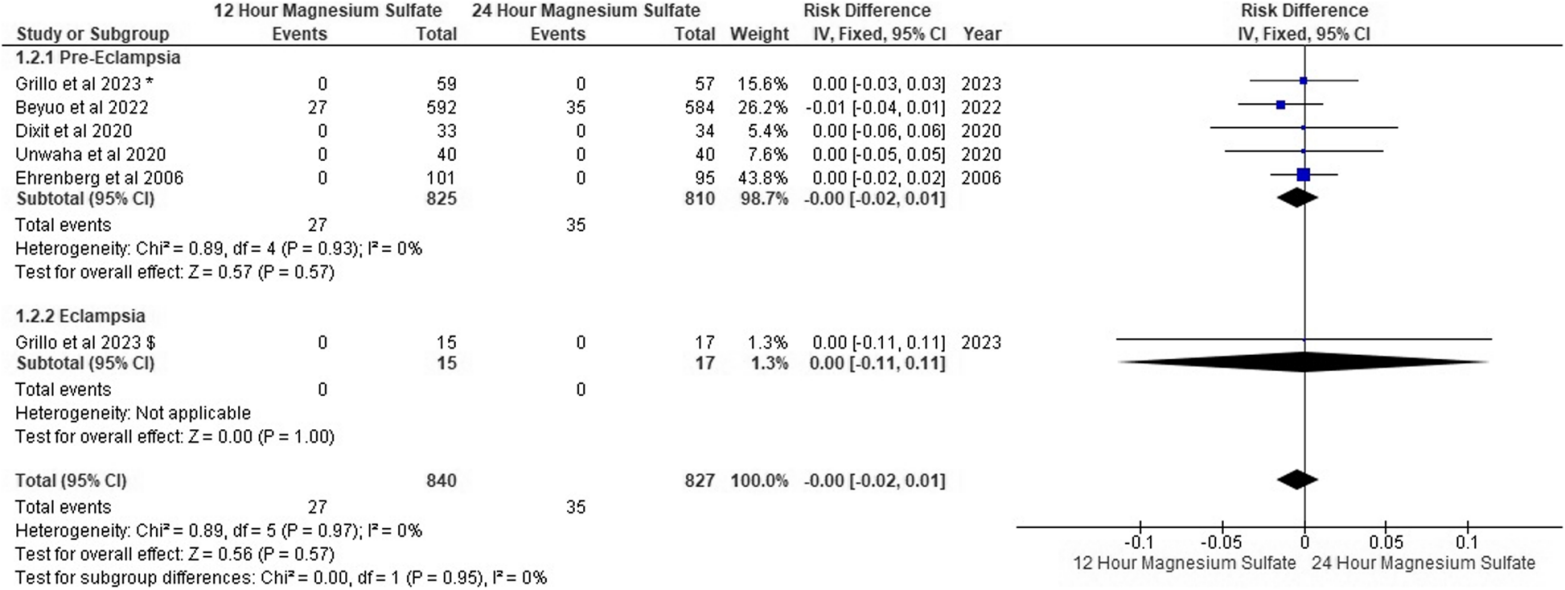
Respiratory depression forest plot

We found no respiratory depression risk difference between both MgSo_4_ regimen groups in both pre-eclampsia patients and eclampsia (RD: -0.00, 95% CI [-0.02, 0.01], *P* = 0.57), (RD: -0.00, 95% CI [-0.11, 0.11], *P* = 0.57), respectively and the pooled results were homogeneous (*P* = 0.93, I^2^: 0%), (*P* = 0.97, I^2^: 0%) as shown in Fig. 7.

### Pulmonary edema

**Fig. 8:**
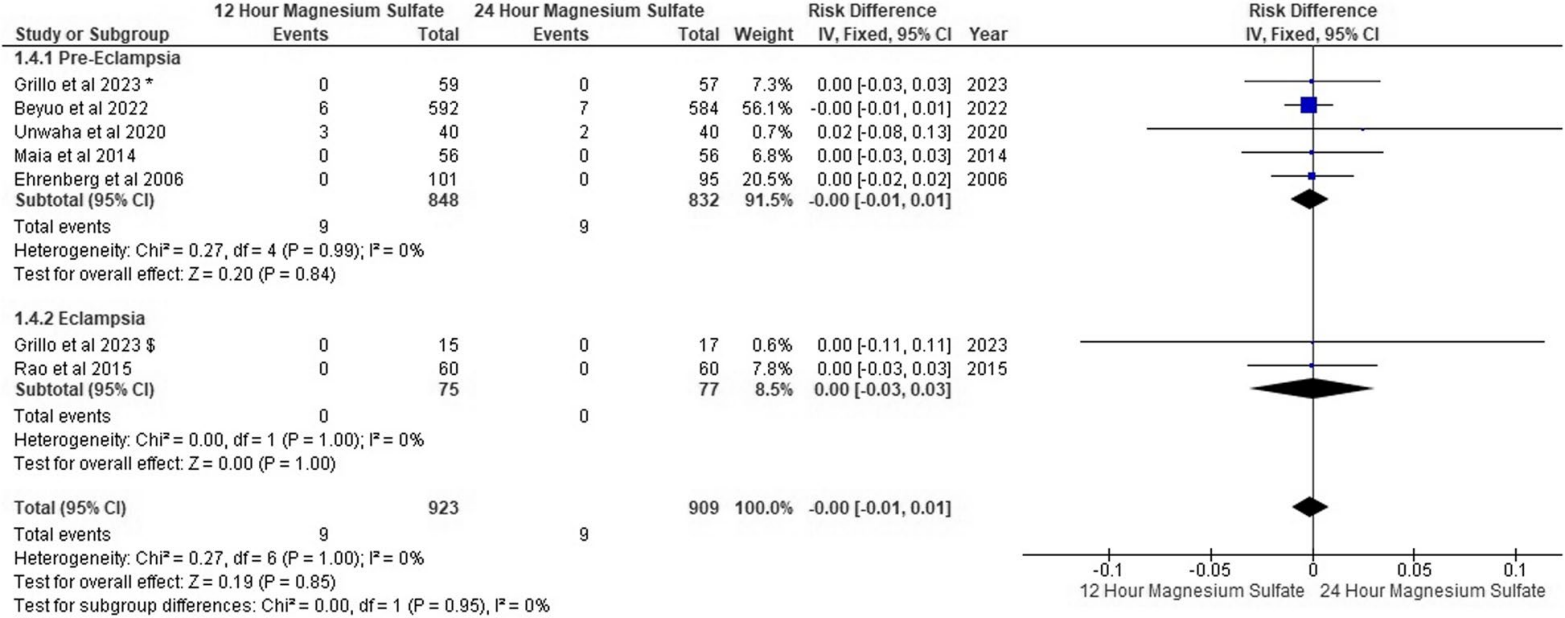
Pulmonary Edema forest plot

In the pre-eclampsia subgroup, nine patients in the 12-h MgSo_4_ group suffered pulmonary edema, and nine in the 24-h MgSo_4_ group. In the eclampsia subgroup, no patients suffered from pulmonary edema in both groups. Overall, both MgSo_4_ regimen groups showed the same number of patients complicated with pulmonary edema with no risk deference (RD: -0.00, 95% CI [-0.01, 0.01], *P* = 0.85). The pooled results were homogeneous (*P* = 1, I^2^: 0%) as shown in Fig. 8.

## Discussion

We conducted this systematic review and meta-analysis to condense the evidence on clinical efficacy and safety of 12-h MgSO_4_ compared to 24-h MgSo_4_ in the prophylaxis and treatment of seizures in pre-eclampsia and eclampsia patients. We included 13 RCTS with 2809 patients (12-h MgSo_4_ = 1435 and 24-h MgSo_4_ = 1374). We found an overall low risk of bias in nine trials, some concern in two trials, and high risk in another two studies. The pooled findings confirmed that there is no statistically significant difference between both regimens in occurrence of seizures, diminished deep tendon reflexes, respiratory depression, and pulmonary edema in pre-eclampsia and eclampsia patients. There is no heterogeneity in all outcomes. There was no evidence of publication bias for occurrence of seizures.

MgSo_4_ is the preferred and standard treatment for managing seizures events in eclampsia patients and as a prophylaxis for pre-eclampsia patients [18]. Convulsions have been observed to occur with greater frequency during or following the first pregnancy, and these episodes have been associated with a poor prognosis [19]. A crucial question remains as to whether they represent epilepsy or specific pregnancy complications [19]. It has been suggested that eclampsia differs from epilepsy as epilepsy is chronic and recurs throughout the lifespan, whereas eclampsia does not [20]. Eclampsia can occur in prepartum, intrapartum, or postpartum [21]. According to our included studies, three studies [1, 3, 13] included patients who developed eclampsia ever prepartum, intrapartum, or postpartum. Khan et al. [17] included patients who developed eclampsia postpartum, while Rao et al. [11] did not specify the period of convulsion occurrence. The studies that included patients with pre-eclampsia did not specify when convulsion occurred as it is considered only as a complication in un/maltreated patients. Our primary outcome is the prevention of the occurrence of seizures in pre-eclampsia patients and its recurrence in eclampsia patients. Our findings showed no difference between both 12-h and 24-h MgSo_4_ groups in the occurrence of seizure (*N* = 8, *N* = 10), respectively, in both pre-eclampsia and eclampsia patients.

Although the MgSo4 effectively treats seizures, there is a risk of developing hypermagnesemia toxicity. The therapeutic level of Mg to maintain its treating effect ranges between 3.5 – 7 mEq/L, and when it exceeds that, toxicity symptoms begin to appear [22]. Affection of deep tendon reflexes represents one of the MgSo_4_ serious toxicity effects. Diminished deep tendon reflex is the first sign of MgSo_4_ toxicity as the patellar deep tendon reflex disappears when the concentration of serum Mg ranges between 8–10 mEq/L [22]. This study found no difference between 12-h and 24-h MgSO_4_ groups in the affection of deep tendon reflexes with (*N* = 36, *N* = 43), respectively, in pre-eclampsia and eclampsia patients.

Respiratory depression is also considered one of the serious MgSo_4_ toxicity signs. It is reported when the level of Mg reaches > 13 mEq/L [22, 23]. Based on our analysis findings, there is no difference between 12-h and 24-h MgSO_4_ groups in the occurrence of respiratory depression with (*N* = 27, *N* = 35), respectively, in both pre-eclampsia and eclampsia patients.

Pulmonary edema may present in patients by shortness of breath, cough, chest pain, and frothy sputum [24]. The analysis of our study showed no difference between both 12-h and 24-h MgSO_4_ groups in the occurrence of respiratory depression with (*N* = 9, *N* = 9), respectively, in both pre-eclampsia and eclampsia patients.

Although Sullivan et al. [25] is the last systematic review that published and discussed a similar comparison, it compares 24-h MgSO_4_ and less than 24-h MgSO_4_. So, it is difficult to compare our results to Sullivan et al. [25] as they did not specify the duration of therapy in the intervention group. Additionally, Yifu et al. [26] also compare less than 24-h MgSO_4,_ which includes 12-h MgSO_4,_ and other regimens with 24-h MgSO_4_, so dependently, it cannot be compared to our study. Upon that, our study is considered to be the first systematic review and meta-analysis that compares only the 12-h MgSO_4_ with 24-h MgSO_4_ therapy with more detailed data and analysis of signs of toxicity of MgSO_4_.

Our study had many strengths; it is considered the first systematic review and meta-analysis to include patients with eclampsia, severe pre-eclampsia, and mild pre-eclampsia. All our 13 included studies were RCTs. We updated the search on PubMed and included the last RCT published in March 2023. This study analyzed data from 2809 patients and performed a subgroup meta-analysis on whether patients were diagnosed with eclampsia or pre-eclampsia in all reported outcomes.

However, there were also some limitations, our sample size was 2813 while only 2809 patients were included in the analysis as there were four patients excluded from the analysis in Anjum et al. [3] and accordingly excluded from our study analysis. Beyuo et al. [1] included both patients of pre-eclampsia and eclampsia without separating their data; we preferred including it in the pre-eclampsia subgroup, as 90.1% of its patients were diagnosed as patients with pre-eclampsia. Only English papers were included because we did not have access to a reliable method to translate non- English papers. Ehrenberg et al. [10] with mild pre-eclampsia patients is included in our pre-eclampsia subgroup, so we could not conduct a subgroup while other studies represent patients with severe pre-eclampsia.

We totally recommend establishing more clinical trials in industrialized countries as we only found one paper from the USA. We couldn’t compare between the two periods of MgSo_4_ regarding the cost effectiveness because there are not any included studies discuss the cost effectiveness of the two period of MgSo_4_. So that, we recommend the future research to compare between the regimes regarding the cost and to be powered enough to investigate the safety of both regimens.

## Conclusion

MgSO_4_ is the most effective anticonvulsant for prophylaxis and treatment of fits in patients with pre-eclampsia and eclampsia, considered serious medical conditions affecting pregnant females. Our Systematic review and meta-analysis showed no significant difference between the 12-h and 24-h regimens in the occurrence or recurrence of seizures, respiratory depression, absence or decrease of deep tendon reflex, and pulmonary edema.

## Data Availability

Data will be provided upon request from Rahma Sameh Shaheen (rahma193226@fmed.bu.edu.eg).

## Abbreviations

DBP: Diastolic blood pressure
IM: Intra-muscular
IV: Intra-venous
MgSO_4_: Magnesium sulphate
RCTs: Randomized controlled trials
SBP: Systolic blood pressure
USA: United States of America
